# Addressing Underscreening for Cervical Cancer among South Asian Women: Using Concept Mapping to Compare Service Provider and Service User Perspectives of Cervical Screening in Ontario, Canada

**DOI:** 10.3390/curroncol31110498

**Published:** 2024-11-01

**Authors:** Kimberly A. Devotta, Patricia O’Campo, Jacqueline L. Bender, Aisha K. Lofters

**Affiliations:** 1 Dalla Lana School of Public Health, University of Toronto, Toronto, ON M5T 3M7, Canada; 2 Peter Gilgan Centre for Women’s Cancers, Women’s College Hospital, Toronto, ON M5S 1B3, Canada; 3 MAP Centre for Urban Health Solutions, St. Michael’s Hospital, Unity Health Toronto, Toronto, ON M5B 1W8, Canada; 4 Department of Supportive Care, University Health Network, Toronto, ON M5G 2C4, Canada; 5 Department of Family and Community Medicine, University of Toronto, Toronto, ON M5T 3M7, Canada

**Keywords:** concept mapping, cervical screening, health equity, women’s health, community engagement, patient–provider interactions, culturally tailored interventions

## Abstract

Cervical cancer is largely preventable through screening and treatment of cervical lesions. In the province of Ontario, South Asian women have some of the lowest rates of screening. The roles of service providers—those in healthcare and community services—and their interactions with screen-eligible people can greatly impact the uptake of screening. In our study, we used concept mapping (CM) to engage over 70 South Asian service users (i.e., those eligible for cervical screening) and service providers to identify a range of ideas and experiences that impact uptake of cervical screening for South Asian women, which were then rated by 45 participants in terms of ‘importance’ and ‘ease to address’ to encourage participation in cervical screening. Overall, ideas related to knowledge and education were rated as most important and easiest to address by both groups. Some differences were seen with South Asian service users valuing the importance of addressing ‘cultural beliefs and influences specific to sexual health’ more than service providers, while service providers valued the importance of addressing ‘lack of comfort and supportive relationships’ more than South Asian service users. Future interventions should target the knowledge and education needs of service users and increase service providers’ awareness of cultural beliefs and influences specific to sexual health.

## 1. Introduction

Participating in cervical screening has great potential to reduce a person’s risk of developing cervical cancer. Through efforts of organized screening programs, including evidence-based guidelines on initiation, intervals, and cessation, as well as reminders for continued screening, the potential to decrease and even eliminate cervical cancer is promising. However, programs such as the Ontario Cervical Screening Program (OCSP) have experienced a plateauing of participation rates since its introduction in 2000 [1], with certain subgroups, such as immigrants and refugees, having disproportionately lower rates [2]. The OCSP currently sends out invitation letters for people who are eligible and due for screening and has published a pamphlet with cervical-screening information.

Currently, the World Health Organization and other bodies, such as the Canadian Partnership Against Cancer, have set the goal to eliminate cervical cancer by 2040 [3,4]. One of the strategies to achieving this is to increase participation in cervical screening. Currently in Ontario, cervical screening is primarily carried out using a Papanicolaou (‘Pap’) test, which is most often completed by a primary care provider, but the province will very soon make a major change and implement HPV testing which uses a swab that can be clinician- or self-collected [5,6].

Understanding and improving cervical screening is important for the health of people with a cervix. Work conducted in this area has shown that South Asian women have some of the lowest rates of screening in Ontario [7,8,9]. Additionally, with restrictions on healthcare services during the COVID-19 pandemic, including a pause to screening, these inequities are projected to be exacerbated for many groups in the province [10].

The relationship between, and viewpoints and experiences of, South Asian service users (i.e., South Asian people eligible for cervical screening) and service providers (i.e., people in healthcare and community services, such as primary care providers, health promoters, and settlement workers) may be particularly important in the uptake of cervical screening. Interactions with primary care providers can be one of the most critical influences impacting the uptake of screening for South Asian women. Physicians can be seen as authoritative and trustworthy, and if they do not recommend screening, women may not think it is important [11]. Past experiences of feeling rushed, feeling unheard, and painful examinations can discourage further screening [12,13]. At the community level, participation in cancer screening can be improved by the presence of educational campaigns and other informative media that convey information about screening frequency, procedures, and locations [14,15,16]. However, stigma within certain South Asian ethnic communities around sexual activity, virginity, and marital status can also pose barriers, as a woman getting screened would imply certain details about their sexual activity [17,18,19,20].

In Ontario, Pap tests are often conducted in a doctor or nurse’s office by primary care providers and sometimes in public health units, sexual health clinics, and community health centers. Initiatives in the community around health promotion, newcomer settlement, peer support, and other programming also make people working in community services a necessary part of cervical screening due to their influence in spreading awareness and providing support to encourage cervical screening [21]. South Asian service users may differ from service providers in what barriers to cervical screening they view as important or easy to address, as they hold different levels of power and privilege in the community, and their perspectives would provide important context for understanding current rates of underscreening and direction for ways to encourage an uptake in cervical screening. Additionally, evidence of biased attitudes has been shown amongst healthcare providers in the forms of implicit and explicit attitudes and stereotypes [22], and addressing these amongst service providers involved in the delivery of cervical screening for South Asian service users can be critical.

In our study, we set out to understand how the lives and experiences of South Asian women shape their decisions around cervical screening. We chose to use concept mapping (CM), as it is a collaborative method that brings multiple stakeholders together to develop a common framework that can be used for planning [23]. Stakeholders come together to brainstorm ideas, and then structure and rate them to reflect their thinking and prioritization for action. In an earlier paper, we demonstrated how a range of experiences impact cervical screening and how participants thought these experiences relate to each other and cluster into six larger themes: (i) personal beliefs and misconceptions around cervical screening; (ii) education and knowledge issues around cervical cancer; (iii) cultural beliefs and influences specific to sexual health; (iv) barriers to prioritizing uptake of cervical screening; (v) system/infrastructure gaps or inadequacies; and (vi) lack of comfort and supportive relationships in healthcare [24]. In this issue, we have published another paper that looks at specific action items for addressing cervical screening, based on rating data from the group [25].

In this paper, we present a sub-group analysis to demonstrate how different stakeholders value the larger identified themes (from a previous analysis), specifically regarding the importance of the issue of improving screening participation and the ease of addressing the issue of improving screening participation. This paper is guided by the question: when it comes to making decisions around cervical screening, how do participants value the identified themes, and are there similarities and differences between South Asian service users and service providers participant sub-groups?

## 2. Materials and Methods

### 2.1. Ethics

This study was approved by the Research Ethics Board at the University of Toronto (REB# 43281).

### 2.2. Study Design

We used a concept-mapping (CM) study design to engage different stakeholder groups (i.e., South Asian service users and service providers) to build a conceptual framework that reflects how they view cervical screening amongst South Asian women. CM was developed by Kane and Trochim [23] in the 1980s and is designed to collect ideas from a group of individuals, identify and use graphics to represent how they organize the interrelationship amongst the ideas, and to represent their group thinking. Work carried out by Burke and colleagues [26] brought CM into public health research and emphasized the participatory aspects of this method for engaging people in research. CM is a participant-driven method where participants brainstorm ideas around a specific topic and then independently rate and sort the ideas to show how they value and relate to the ideas. The result of this method is a conceptual framework that reflects how a group views a particular topic [27]. CM has four main participant activities. In brainstorming, participants generate ideas related to a defined focal prompt. Sorting and rating take place simultaneously, and by the end, participants have organized and prioritized the data that came out of the brainstorming activity. Finally, in the map interpretation activity, participants are brought together to review, discuss, and potentially challenge the results of the CM analysis. We have previously written detailed papers on the brainstorming and sorting activities [24,25], which we describe in brief for context in Section 2.5.1: Brainstorming and Sorting to Create Thematic Clusters. This paper focuses on the analyses of the rating activity data to better understand how different stakeholders valued the themes that emerged from the CM process.

### 2.3. Participants

A purposive sampling strategy was used for heterogeneity in the participant sample. CM does not require a statistically significant sample size. Many of the activities are qualitative in nature, so a large sample size is not needed. In CM, a broad set of ideas is valued over a representative sample, and therefore non-random sampling is preferred [23]. We engaged a range of stakeholders that included (i) South Asian women living in the Greater Toronto Area (GTA), (ii) community champions—trusted female members of the South Asian community with pre-existing connections with local community groups and organizations, (iii) people who work in organizations that serve South Asian women in the GTA, and (iv) healthcare providers serving South Asian patients. Participants were not obligated to participate in every CM activity, nor was participation in previous activities required to take part in later activities. In each round, additional participants were recruited, with the exception of map interpretation.

The eligibility criteria for South Asian women included that they are or have ever been eligible for cervical screening in Ontario (21 years of age or older, has a cervix, and has been sexually active) and self-identify as South Asian. In our study, we considered ‘South Asian’ to refer to ancestry and not simply place of birth. This includes people who can trace their origins back to countries in South Asia, such as: Afghanistan, Bangladesh, Bhutan, India, Maldives, Nepal, Pakistan, Sri Lanka. All other participants from healthcare and the community had to identify as being in a role that works or serves South Asian women. This included healthcare providers, social service workers, health promoters, community organizers, and others in the healthcare and social services. These participants were also asked to gauge their familiarity with cervical cancer screening amongst South Asian women. All participants had to be at least 18 years of age and speak conversational English.

With the assistance of a community champion—a South Asian woman with many social and professional connections with South Asian groups, programming, and other individuals—participants were recruited through word-of-mouth and posters that were distributed via social media and organizations with a large South Asian client population. Additional efforts were made for service providers, including email distribution lists and hospitals, primary care teams, and other organizations in the community.

### 2.4. Demographics

Demographic questions were asked before each CM activity, at the start of the activity to ensure participants did not submit the task without completing the questions. Each participant was asked to select the response that best described themselves: (i) I identify as South Asian; (ii) I work in a role or an organization that serves South Asian women and I identify as South Asian; (iii) I work in a role or an organization that serves South Asian women and I do not identify as South Asian; (iv) I work as a primary care provider and I identify as South Asian; or (v) I work as a primary care provider and I do not identify as South Asian. The first category was considered ‘South Asian service users’, and the remaining categories are referred to as ‘service providers.’ These became our stratification variables for the pattern match analysis that is described below (Section 2.6.2). Service providers were also asked how long they have been in their area of work, and South Asian service users were asked if they had ever had a Pap test. All participants were asked their age and gender identity. In the sorting, rating, and map interpretation activities (described below), service providers were additionally asked for the approximate percentage of South Asian people that they serve, and further details about their roles in healthcare and the community.

### 2.5. Concept-Mapping Activities

**Figure 1:**
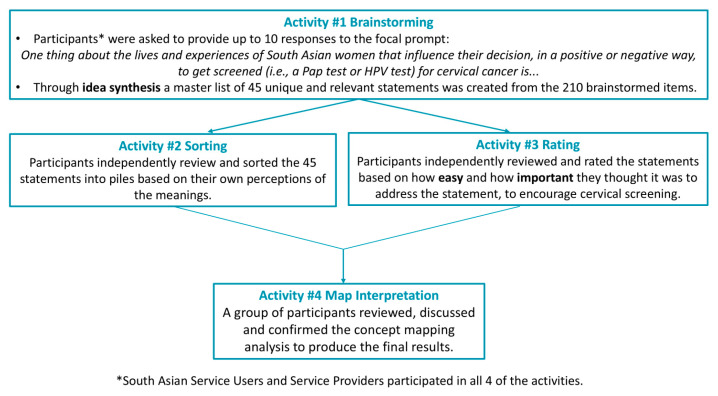
Summary of concept mapping activities. South Asian service users and service providers participated in each of the 4 activities.

Prior to starting the CM activities, the study team, community champion, and other South Asian women finetuned the wording of the CM prompt to be: ‘One thing about the lives and experiences of South Asian women that influence their decision, in a positive or negative way, to get screened (i.e., a Pap test or HPV test) for cervical cancer is…’ Determining the wording of the prompt is an important first step in the CM process. Once this was established, the brainstorming, sorting, rating, and map interpretation activities were conducted. The software Concept Systems Group Wisdom (Version 2013.322.11 Ithaca, NY, USA) was used to collect, store, and analyze the CM data. Participants were able to complete brainstorming, sorting, and rating online. Some participants completed rating and sorting in-person, so they could obtain real-time assistance—including clarifications on the instructions and how to record their responses—from a member of the research team. Map interpretation was carried out over zoom as a group. Data collection took place from September 2022 and August 2023. Participants were given an e-gift card upon completion of each activity: $30 for brainstorming, $40 for sorting, and $30 for map interpretation. The four concept-mapping activities are summarized in Figure 1.

#### 2.5.1. Brainstorming and Sorting to Create Thematic Clusters

Details of the brainstorming and sorting have been previously reported [24,25]. Briefly, in the brainstorming activity, participants were asked to provide up to 10 responses to the CM prompt. Using an anonymous weblink, participants were first asked to complete the demographic questions and then review the already collected brainstorming responses. If participants had additional ideas to add, they could do so, and if not, they could submit the activity without adding any new ideas. Participants could come back to the activity multiple times while it was open but had to submit demographic responses each time. Once the activity closed, members of the research team reviewed and reduced the ideas to create a manageable list of unique and on-topic statements, creating the master list to be used in the rest of the activities.

During the sorting activity, participants independently reviewed the master list and provided their perceptions on the similarity between the items and how they view the interrelationship of the ideas [23]. Participants did this by sorting the items in piles that made sense to them, without leaving any one item on its own or creating a catchall pile. Each statement could only be sorted one time, so participants were encouraged to place it in the pile they felt the strongest about. Participants that completed this task online were given personalized login information so they did not have to complete it all at once.

Findings from the application of hierarchical cluster analysis to the sorting data enabled the generation of cluster maps [23]. Hierarchical cluster analysis groups individual statements into clusters of statements that reflect similar concepts [28]. The cluster map displays how the items were sorted and thought about thematically. During the map interpretation activity, participants named these clusters to reflect the statements within them: (i) personal beliefs and misconceptions around cervical screening; (ii) education and knowledge issues around cervical cancer; (iii) cultural beliefs and influences specific to sexual health; (iv) barriers to prioritizing uptake of cervical screening; (v) system/ infrastructure gaps or inadequacies; and (vi) lack of comfort and supportive relationships in healthcare.

#### 2.5.2. Rating Activity to Understand Valuing of Statements and Themes

The rating activity presented participants with the master list that came out of the brainstorming activity and asked them to rate each statement based on two rating prompts. The first question used a 5-point Likert scale from ‘strongly disagree’ to ‘strongly agree’ and asked, ‘how much do you agree or disagree that removing or fixing this barrier would improve cervical cancer screening participation amongst South Asian women?’ (‘importance’). The second question similarly used a 5-point Likert scale that ranged from ‘very difficult to solve or address’ to ‘very easy to solve or address’ and asked participants, ‘how easy do you think it is to solve or address this issue so that South Asian women will be encouraged to participate in cervical cancer screening?’ (‘ease’). The sorting and rating activities took place at the same time, meaning one activity did not have to be closed before the other one could be started. Participants that did both activities started with the sorting activity and then did the rating activity. Kane and Trochim [23] recommend that sorting take place first, as it encourages participants to think though the semantic similarities between the statements. For those who completed the activities online, these instructions were provided to them over email and in the order of the activities when they logged into their personalized weblinks. For those who attended the in-person event, the materials were presented to them one activity at a time.

### 2.6. Data Analysis

#### 2.6.1. Valuing of the Clustered Themes

To understand how the group valued the importance and ease of addressing the clustered themes, an average rating was calculated for each statement and then for each cluster. These values can be overlaid on the cluster map to create a cluster rating map.

#### 2.6.2. Similarities and Differences Within Stratification of South Asian Service Users and Service Providers

While the average cluster ratings show us what clusters of statements the participants thought were most important and easy to address, it is also critical to explore how these perspectives of importance and ease to address may differ amongst subgroups. It is important to remember that while all the statements and clusters are important and, to some degree, can be addressed, the rating questions can show us what participants think are of particular importance and of particular ease to address. To further understand the valuing of the clustered themes, we used pattern matching to identify where opinions amongst South Asian service users and service providers may diverge. A pattern match is a pair-wise comparison of cluster ratings across two variables [28]. Here, we used it to visually give us a side-by-side comparison of how two stakeholder groups rated the statement clusters for each rating question. Pattern matching is an important technique in CM to show differences and similarities amongst stakeholder groups, an important area to understand when planning to address the issue that is being studied [23]. Understanding these similarities and differences is important for planning guidance to address issues around cervical screening, especially for strategies and interventions tailored to different South Asian service users or service providers.

In a pattern match, a ladder graph is used to represent how the average cluster ratings compare between two variables. The vertical axes display the range of average cluster values. Each axis represents a variable or participant subgroup, depending on the purpose of the comparison. We used a relative scale for the pattern matches presented here, where the vertical axis represents the highest to lowest average cluster ratings.

The pattern matches were computed using the software GroupWisdom (Version 2013.322.11 Ithaca, NY, USA). In the pattern match display, the vertical axis on each side of the ladder graph represents a different participant group (i.e., South Asian service users and service providers). All 6 of the clusters are listed on each axis, and the position of each cluster is based on the average cluster-rating value for the rating question that the pattern match represents. Lines connect the same cluster on each side of the ladder graph, from the position where it has been placed [23]. The slope of the line depicts the relationship between what is being compared, where the steeper the slope is, the greater the difference [23]. A correlational value called the Pearson product–moment correlation is calculated for the pattern match ladder graph to describe the relationship between the two variables being compared in the graph [23]. This value ranges from −1.00 to 1.00, and the direction of the line—positive or negative—indicates if the relationship between the two variables is inverse or aligned [28]. If there is a perfect correlation between the two patterns being compared, there will be a straight line between all clusters, similar to the rungs on a ladder [23]. Pattern matches can use a relative or absolute scale. For the purposes of our study, we chose a relative scale which places the high and low average values on the vertical axes specific to each rating variable that is being compared, in this case, importance and ease [28]. This allows for the display of subtle variations in the order of ranking on each vertical axis representing South Asian service users and service providers. With this type of scale, we would more clearly see similarities (i.e., straight lines) and differences (i.e., sloped lines) in how each subgroup’s average cluster ratings ranked each cluster.

#### 2.6.3. Map Interpretation

During the map interpretation activity, participants attended a group session where they were presented with results from each CM activity (brainstorming, sorting, and rating). This included a presentation of the cluster-rating maps and pattern match ladder graphs by the group facilitator (KD). This was used as a basis for a group discussion about areas and differences [23] amongst participant sub-groups, including the comparison of South Asian service users and service providers. Participants also discussed whether there was anything confirming or surprising in the overall cluster ratings.

## 3. Results

### 3.1. Participant Sample

Based off the collected demographics, between 44 and 72 participants completed the brainstorming activity. To make participation as low-barrier as possible, we used an anonymous link that participants could use without setting up login information. Due to this, we do not know how many of the 72 times that the link was accessed were people who came back multiple times to add in responses and were therefore asked to complete demographic questions again. From participants who submitted the activity and completed the second weblink to choose their honorarium, we know that there were at least 44 unique participants. The sorting activity was completed by 22 participants (11 South Asian service users and 11 service providers), rating by 45 participants (25 South Asian service users and 20 service providers) and map interpretation by 9 participants (4 South Asian service users and 5 service providers). In the analysis, 18 participant-sorting datasets were used, as 4 of them were excluded for different errors in their sorting (e.g., sorted less than 75% of statements, statements were sorted multiple times). Additionally, 1 participant in each of the rating questions had their data excluded because they had used the same rating value for all their responses to that question; therefore, 44 participant-rating datasets were included in the analysis of each rating question.

The collected demographics are presented below. Table 1 summarizes the demographics collected during the brainstorming activity, while Table 2 and Table 3 present the demographics for the sorting, rating, and map interpretation activities, divided by South Asian service users (Table 2) and service providers (Table 3). Since the brainstorming activity was performed anonymously, the related demographics cannot be disaggregated and are presented in Table 1.

Aside from one participant in the brainstorming activity and two participants in the rating activity, all the participants in the service provider category also identified as South Asian. All participants, except for one, in the brainstorming round identified as female. Most participant ages were within the range of 31 to 50. Most participants who were asked about having a Pap test had completed one before. Almost all service providers who worked in healthcare and the community had been doing so for at least 6 years. Service providers also indicated that 9% to 85% of the population they serve is South Asian. Participating service providers represented a range of positions within healthcare and the community. While more than half of the service providers who participated in rating were healthcare providers, we had originally aimed to get even more primary care providers, as they are often the ones who perform Pap tests in the province of Ontario.

### 3.2. Rating Values for Clusters to Understand How Participants Thought About the Themes in Terms of Importance and Ease to Address

**Figure 2:**
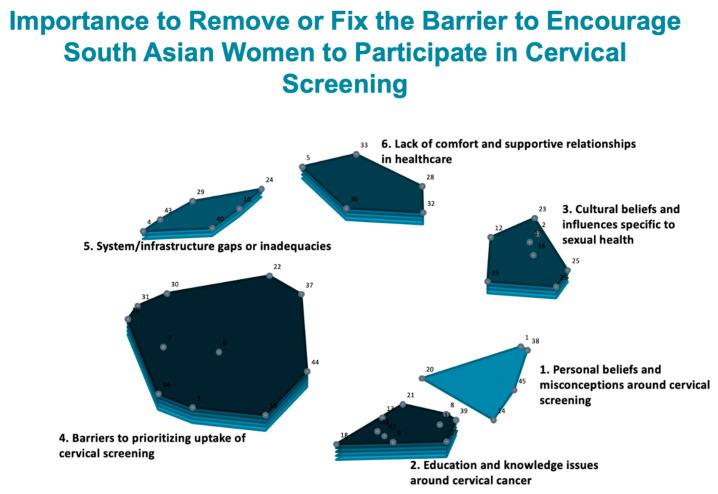
Cluster-rating map showing the average cluster ratings for ‘importance’, indicated by the number of layers.

**Figure 3:**
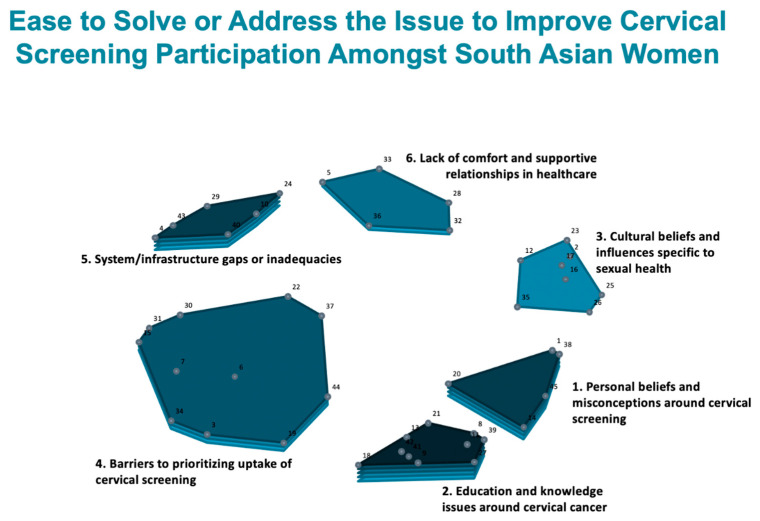
Cluster-rating map showing the average cluster ratings for ‘ease to address’, indicated by the number of layers.

Table 4 shows a summary of the average cluster-rating values for ‘importance’ and ‘ease to address.’ The average rating values were divided into categories: ‘high’ (average rating 3.8 or higher), ‘moderate’ (average rating 3.7–3.0), and ‘low’ (average rating 2.9 or lower). This shows us the averaged ratings from the 45 South Asian service users at the cluster level. These data are overlayed onto the cluster map to create the cluster-rating maps presented in Figure 2 and Figure 3. In these maps, layers reflect average rating where more layers mean a higher average rating is given to the cluster in terms of ‘importance’ (Figure 2) and ‘ease to address’ (Figure 3).

All the clusters were, on average, rated high for importance and comparatively low for ease to address. The highest-rated cluster for both rating questions was ‘Cluster 2: education and knowledge issues around cervical cancer’ (‘high’ for importance; ‘moderate’ for ease to address; 5 layers). Two other clusters, ‘Cluster 4: barriers to prioritizing uptake of cervical screening’ and ‘Cluster 6: lack of comfort and supportive relationships in healthcare’, were also rated high for importance (‘high’ and 5 layers; ‘high’ and 4 layers). Of note, ‘Cluster 3: cultural beliefs and influences specific to sexual health’ has a relatively high average rating (4 layers) for importance but a low average cluster rating for ease to address (1 layer). This shows us that cultural beliefs and influences that are specific to sexual health are perceived as amongst some of the most important factors impacting uptake of cervical screening, but they are also seen as the hardest to address for the purposes of increasing screening participation. Similarly, while the cluster ‘lack of comfort and supportive relationships in healthcare’ had a high average rating for importance, it was also one of the lowest-rated clusters for ease to address (2 layers).

**Figure 4:**
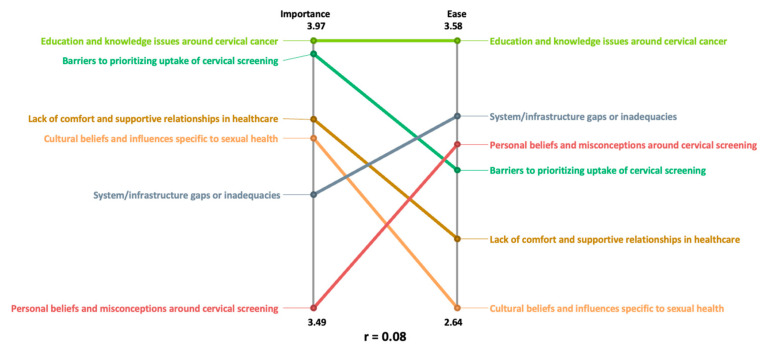
Relative pattern match comparing average cluster ratings ‘importance’ (*n* = 44) and ‘ease’ (*n* = 44). These rating variables measure how important and easy to address the statements were to encourage cervical screening.

Figure 4 depicts a relative scaled pattern match between the rating variables: importance (*n* = 44) and ease to address (*n* = 44). For the most part, more clusters are rated higher for importance than for ease to address. The Pearson product–moment correlation coefficient, r = 0.08, indicates a very weak relationship between the two variables. The two variables are not considered related, and this further supports our decision to look at them separately for the pattern matches below. These results begin to show that some of the most important aspects that impact cervical screening participants are also some of the most difficult to address.

### 3.3. Using Pattern Matches to Understand the Importance- and Ease-Rating Differences and Similarities Amongst South Asian Service Users and Service Providers

**Figure 5:**
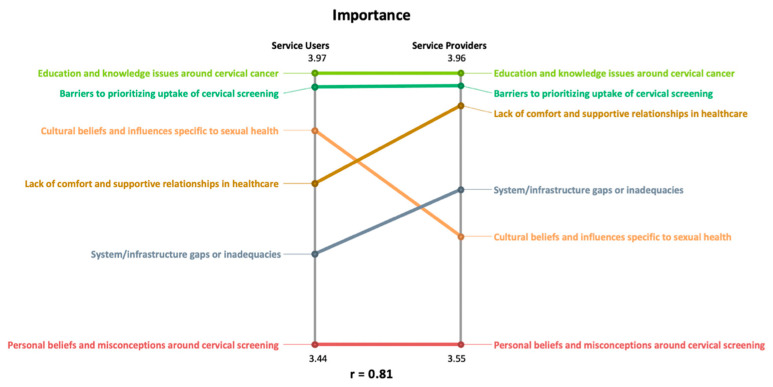
Relative pattern match comparing average cluster ratings for how South Asian service users and service providers rated each statement for the question: how much do you agree or disagree that removing or fixing this barrier would improve cervical cancer screening participation amongst South Asian women?’.

Of the 44 participants whose rating data were used in the pattern match, 24 of them identified as South Asian service users and 20 of them were service providers. The pattern matches presented in this results section show a comparison between these subgroups of 24 and 20 participants. Figure 5 shows the comparison of average cluster importance ratings between South Asian service users and service providers. Overall, we can see that there is a strong correlation between South Asian service users and service providers ratings of importance (r = 0.81). South Asian service users and service providers agree quite strongly on the importance of addressing the statements in Cluster 2: education and knowledge issues around cervical cancer and Cluster 4: barriers to prioritizing uptake of cervical screening, as depicted by their rank placement on the axis and straight line. For both groups, these were their top-two rated clusters with very similar average ratings. We also see agreement around Cluster 1: personal beliefs and misconceptions around cervical screening, which was rated the lowest.

Depicted by the lines with a steeper slope, we can see areas of difference between the two groups. The importance of addressing Cluster 3: cultural beliefs and influences specific to sexual health, was ranked high amongst South Asian service users and much lower for service providers. Comparatively, service providers viewed addressing Cluster 6: lack of comfort and supportive relationships in healthcare and Cluster 5: system/infrastructure gaps or inadequacies, as more important to address than South Asian service users rated those clusters.

**Figure 6:**
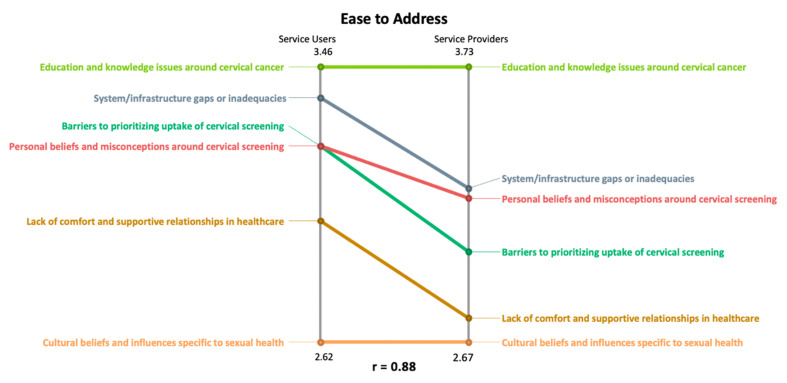
Relative pattern match comparing average cluster ratings for how South Asian service users and service providers rated each statement for the question: how easy do you think it is to solve or address this issue so that South Asian women will be encouraged to participate in cervical cancer screening?

Figure 6 displays a comparison of South Asian service users to service providers around their average cluster ratings for the rating question around how easy participants believe it is to address or solve the issue represented by the statement, to encourage South Asian women to participate in cervical screening. The correlation coefficient (r = 0.88) tells us that there is a strong relationship between South Asian service users and service providers and how they view the ease of addressing the statements. For the most part, South Asian service users and service providers agreed on the ranking of most of the clusters. Similar to the importance rating, Cluster 2: education and knowledge issues around cervical cancer was considered the easiest to address. Both groups agreed that Cluster 3: cultural beliefs and influences specific to sexual health was the hardest to address. While the remaining clusters were mostly ranked the same between the two subgroups, it is important to point out that South Asian service users perceived them to be relatively easier to address or solve compared to service providers. This is especially so for Cluster 5: system/infrastructure gaps or inadequacies and Cluster 6: lack of comfort and supportive relationships in healthcare.

During the map interpretation session, participants discussed the ranking of Cluster 3: cultural beliefs and influences specific to sexual health, both being very important but hardest to address. Participants discussed how this was not surprising to them, sharing examples of how conversations around sexual health and cervical screening are difficult to have within their families and, in some cases, are nonexistent. Participants also spoke about the great need for more education and opportunities to learn about cervical screening. Two participants even discussed the format of the map interpretation session (i.e., discussion led by a South Asian woman involved in healthcare with all South Asian women in attendance) as a comfortable and effective way to learn and ask questions. Participants seemed to value the work and role of supportive relationships (e.g., friends, family members, peers, healthcare providers) as having a positive impact on South Asian women pursuing screening. Overall, participants at the map interpretation felt the pattern match was in line with what they were thinking, especially around the disconnect between South Asian service users and service providers.

## 4. Discussion

Through concept mapping, we were able to engage South Asian service users and service providers to understand their perspectives about how important and easy it is to address aspects of people’s lives and experiences, in order to encourage cervical screening. Using cluster ratings, we were able to identify some of the top areas that both groups valued, and with pattern matches, we were able to see in which areas people differed. Overall, the area of knowledge and education was rated as most important and most easy to address. Amongst the other clusters, we saw that some of the most important aspects that impact cervical screening amongst South Asian women are also rated as the most difficult to address. This was especially so for the cluster of statements about ‘cultural beliefs and influences’ and ‘lack of comfort and supportive relationships.’ The stratification of the data by South Asian service users and service providers shows us that, overall, there is a strong correlation between how the two groups rated the statements, but some differences occurred, with South Asian service users valuing the importance of addressing ‘cultural beliefs and influences specific to sexual health’ more than service providers, while service providers valued the importance of addressing ‘lack of comfort and supportive relationships’ higher than South Asian service users. These findings provide insight into areas for addressing low rates of cervical screening in the province. These areas of difference can provide important context for understanding current rates of underscreening, as service providers—which includes physicians and nurses—may be underestimating how important cultural beliefs and influences around sexual health are to South Asian service users and their decisions around cervical screening. This also shows us that aspects of healthcare that directly involve service providers, such as relationships in healthcare, as well as gaps and inadequacies, may be more important to service providers because they have more power in that area than they do for cultural barriers.

### 4.1. Addressing Current Issues Related to Underscreening for Cervical Cancer

In our study, knowledge and education around cervical screening was a highly valued area across service users and service providers both in terms of how important it is and how easy it is to address. Current rates of under- or never-screened people have been in part due to unmet information needs about cervical cancer [14]. There need to be strategies to increase knowledge and education around what cervical cancer is, what screening involves, and the impact of screening. This will be even more important in the coming years as major changes to cervical-screening procedures will occur in the province with the introduction of HPV testing.

One of the biggest areas of disagreement between South Asian service users and service providers in the study seemed to be around how important service users found cultural influences around sexual health to be, compared to service providers. This is an important finding, given the studies that have shown provider language skills and sensitive approaches to topics of sexual health, reproductive organs, and cancer factor into screening decisions [15,18,29,30,31]. Cultural beliefs and influences around sexual health can impact how comfortable South Asian service users are to openly discuss and participate in an intimate procedure such as a Pap test. If providers are uninformed or underestimating what and how important the influence of culture is, they can be impacting and limiting access to care.

The theme that emerged as most important to service providers (lack of comfort and supportive relationships) further underscores the need for peers and others that can provide this level of support for South Asian women. In 2012, the Cancer Awareness: Ready for Education and Screening (CARES) program was established in Toronto, Ontario as an intervention to increase knowledge and screening for cervical and breast cancer among newcomers and other marginalized women in the city [2]. This program was highly successful in getting people screened by engaging community agencies and peer leaders. CARES also involved language-specific group education sessions co-facilitated by peers in community settings that were familiar to participants and facilitated access to screening (i.e., transportation, accompaniment) [2]. This program no longer exists due to loss of funding, but its results show the effectiveness of having peer educators and community-based approaches. Overcoming language and cultural barriers through using settings that people are familiar with, and peers that are racially or ethnically similar, can have positive effects on screening participants [2]. These current findings support the efforts of CARES and similar programs and emphasizes the need to establish and stably support such programs for tackling current rates of underscreening and future major changes to cervical screening in Ontario.

### 4.2. Limitations

One limitation of our study is our inclusion criterion that participants needed to be able to speak conversational English. With the amount of interpretation and group discussion in CM, it is difficult to include multiple languages in the same study, as meanings and related interpretations may not always translate accurately in other languages. As a result, we may have excluded some barriers or facilitators that are specific to non-English service users in Ontario. Additionally, our ratings of importance and ease to address, may have been different from the perspectives of people living in Ontario who do not speak English. Finally, while we were able to stratify based on roles in healthcare (service users vs providers), we were not able to further stratify our service users groups along many areas of diversity, such as ethnicity, religion, age, social class, sexual orientation, education, and marital status.

### 4.3. Future Research

In the analysis presented here, we identified how service users and service providers perceived the importance and ease of each of the thematic areas. Next steps will be to further examine the rating data and identify specific impactful action items to address cervical-screening uptake.

## 5. Conclusions

Using concept mapping, we were able to understand how South Asian service users and service providers perceive the different barriers and facilitators to cervical screening, in terms of importance and ease to address to encourage uptake. Education and knowledge issues around cervical cancer were seen as both highly important and easy to address, for both service providers and service users. This is important given the major changes to cervical screening that will happen in Ontario and other parts of the world, which will require effective knowledge translation and exchange to successfully implement HPV testing and not further exacerbate current issues of underscreening. This work also shows that service providers may be underestimating the impact of cultural beliefs and influences specific to sexual health on cervical screening, and that interventions around this may be needed to make healthcare providers and services more responsive and tailored to those who are under-screened.

## Figures and Tables

**Table 1:** Demographics anonymously collected in the brainstorming activity.

Participant Question	Options	Brainstorming (*n* = 72)
What best describes your role in this study? (options aggregated to reflect analysis categories)	South Asian Service User	52
	Service Provider	20
Have you ever had a Pap test?	Yes	46
	No	5
	Unsure	1
What is your age?	21 to 30	8
	31 to 40	19
	41 to 50	24
	51 to 60	13
	61 to 70	8
Do you identify as	Female	71
	Male	1
	Other	0
If you work in healthcare or in the community, how long have you been in this area of work?	1 to 5 years	5
	6 to 10 years	3
	11 to 15 years	4
	16 to 20 years	2
	20+ years	4

**Table 2:** South Asian service users who participated in sorting, rating, and map interpretation.

Participant Question	Options	Sorting (*n* = 11)	Rating (*n* = 25)	Map Interpretation (*n* = 4)
Have you ever had a Pap test?	Yes	10	20	4
	No	1	5	0
	Unsure	0	0	0
What is your age?	21 to 30	0	1	0
	31 to 40	5	10	2
	41 to 50	5	12	2
	51 to 60	1	2	0
	61 to 70	0	0	0
Do you identify as	Female	11	25	4
	Male	0	0	0
	Other	0	0	0

**Table 3:** Service providers who participated in sorting, rating, and map interpretation.

Participant Question	Options	Sorting (*n* = 11)	Rating (*n* = 20)	Map Interpretation (*n* = 5)
Check all that apply for roles you work in healthcare or in the community	Healthcare Provider Roles	9	16	5
	Community Services Provider Roles	13	23	7
What is your age?	21 to 30	1	2	1
	31 to 40	1	4	0
	41 to 50	6	7	3
	51 to 60	3	4	1
	61 to 70	0	2	0
	Choose not to answer	0	1	0
Do you identify as	Female	11	20	5
	Male	0	0	0
	Other	0	0	0
If you work in healthcare or in the community, how long have you been in this area of work?	1 to 5 years	0	1	0
	6 to 10 years	4	8	3
	11 to 15 years	1	3	1
	16 to 20 years	2	3	0
	20+ years	3	4	1
If you work in healthcare or in the community, what percentage of the population that you serve is South Asian?		9% to 85%	9% to 85%	9% to 85%

**Table 4:** Clusters and statements, with average cluster ratings for ‘importance’ and ‘ease to address’. Cluster numbers are for identification and reference only. They do not reflect a value or rank of the cluster.

Cluster	Statements	Importance Average Cluster Rating Value	Ease to Address Average Cluster Rating Value
Cluster 1: personal beliefs and misconceptions around cervical screening	The belief that you should not “touch” things or go under the knife (meaning any medical procedure) because it brings more harm than good.	moderate (3.49)	moderate (3.22)
	A woman’s belief that cervical cancer screening is not necessary if you have only had one sexual partner.		
	Women may view a Pap test as a dirty procedure where you may bleed afterwards.		
	The belief that if a cervical cancer diagnosis is your fate or destiny, there is no reason to get screened.		
	South Asian women will not get screened because they think they cannot get cervical cancer.		
Cluster 2: education and knowledge issues around cervical cancer	Women believing that a Pap test can lead to an infection	high (3.97)	moderate (3.58)
	A woman’s lack of understanding and education around cervical cancer		
	If a woman believes that cervical cancer is not a severe condition, this can discourage them from getting screened		
	Education about cervical cancer is needed for men in South Asian households		
	Not enough media coverage of cervical cancer screening within the South Asian community		
	Preventative care is not well understood by South Asian women		
	Women believe that if they have an HPV vaccine, they do not need to be screened for cervical cancer		
	Belief that you only have to worry about cervical cancer if you have a problem with your menstruation		
	Women may not know what a Pap test involves		
	Women may not know the purpose of a Pap test		
Cluster 3: cultural beliefs and influences specific to sexual health	Cultural expectations or pressures that the idea of “modesty” prevents women in the South Asian community from getting screened for cervical cancer.	high (3.79)	low (2.64)
	Men in South Asian households make decisions about females getting screened.		
	Negative cultural beliefs behind gynecologist visits leads to South Asian women feeling shame when booking appointments.		
	South Asian women are not comfortable to discuss their sexual history.		
	South Asian women may be worried about their family finding out they are sexually active.		
	Sex is a taboo topic amongst South Asians.		
	Any tests related to sex can be considered dirty.		
	Cervical cancer screening is not openly discussed in the South Asian culture.		
Cluster 4: barriers to prioritizing uptake of cervical screening	Women do not go to the doctor unless they are having an issue.	high (3.94)	moderate (3.13)
	Lack of access to cervical cancer screening information shared by trusted sources.		
	Pap test appointments are viewed as time consuming.		
	Women need reminders to know when they are due for cervical cancer screening.		
	Pap tests can feel painful.		
	Prior negative experience with a Pap test discourages South Asian women from getting screened.		
	South Asian women may prioritize looking after their families over their own health.		
	South Asian women may be too busy with their jobs or careers to take care of their own health.		
	Women are afraid to find out if they have cancer.		
	Women hear other women share negative experiences about getting a Pap test.		
	South Asian women will only get screened when symptoms arise.		
Cluster 5: system/infrastructure gaps or inadequacies	Appointments are not available at times that are convenient for patients.	moderate (3.69)	moderate (3.32)
	Needing to communicate with healthcare providers in English is a barrier for South Asian women to be screened for cervical cancer.		
	Not having a healthcare provider of a similar cultural background makes intimate tests, such as a Pap test, uncomfortable.		
	Foreign-trained physicians may not encourage their patients to do cancer screening, as preventative care may not have been common in their home countries.		
	Family doctor does not encourage cervical cancer screening during appointment.		
	Women do not have a family doctor.		
Cluster 6: lack of comfort and supportive relationships in healthcare	Women do not feel comfortable with their healthcare provider.	high (3.83)	low (2.89)
	Women may be shy to have an examination in that area of their body.		
	Lack of support from family members to go and get screened.		
	Lack of support from friends to go and get screened.		
	Women may be uncomfortable with going to the doctor in general		

## Data Availability

The datasets generated and/or analyzed during the current study are not publicly available due to maintaining the privacy and confidentiality of participants, but are available from the corresponding author on reasonable request.
